# Boosted spatial charge carrier separation of binary ZnFe_2_O_4_/S-g-C_3_N_4_ heterojunction for visible-light-driven photocatalytic activity and antimicrobial performance

**DOI:** 10.3389/fchem.2022.975355

**Published:** 2022-08-05

**Authors:** Shahid Iqbal, Adnan Amjad, Mohsin Javed, M. Alfakeer, Muhammad Mushtaq, Sameh Rabea, Eslam B. Elkaeed, Rami Adel Pashameah, Eman Alzahrani, Abd-ElAziem Farouk

**Affiliations:** ^1^ Department of Chemistry, School of Natural Sciences (SNS), National University of Science and Technology (NUST), Islamabad, Pakistan; ^2^ Department of Chemistry, Government College University, Lahore, Pakistan; ^3^ Department of Chemistry, School of Science, University of Management and Technology, Lahore, Pakistan; ^4^ Department of Chemistry, College of Science, Princess Nourah Bint Abdulrahman University, Riyadh, Saudi Arabia; ^5^ Department of Pharmaceutical Sciences, College of Pharmacy, AlMaarefa University, Riyadh, Saudi Arabia; ^6^ Department of Chemistry, Faculty of Applied Science, Umm Al-Qura University, Makkah, Saudi Arabia; ^7^ Department of Chemistry, College of Science, Taif University, Taif, Saudi Arabia; ^8^ Department of Biotechnology College of Science, Taif University, Taif, Saudi Arabia

**Keywords:** binary heterojunction, synergic effects, photocatalysis, spatial charge separation, antimicrobial activity

## Abstract

A potential method for removing toxins from contaminated wastewater, especially organic pollutants, is photo-catalysis. Here, a simple technique for producing zinc ferrite nanoparticles (ZnFe_2_O_4_ NPS) with varying quantities of sulphur doped graphitic carbon nitride nanocomposites (ZnFe_2_O_4_/S-g-C_3_N_4_ NCs) has been described. Then, using X-ray diffraction (XRD), TEM, EDX, XPS, photocurrent response, EIS, and Fourier Transform Infrared spectroscopy (FT-IR), the photo-catalytic activity of the produced nanoparticles (NPs) and nanocomposites (NCs) was examined and evaluated. The photo-catalytic activity of ZnFe_2_O_4_/S-g-C_3_N_4_ NCs was compared to a model pollutant dye, methylene blue, while degradation was evaluated spectrophotometrically (MB). Solar light has been used through irradiation as a source of lighting. The photocatalytic behaviour of the ZnFe_2_O_4_/S-g-C_3_N_4_ NCs photocatalyst was superior to that of genuine ZnFe_2_O_4_ and S-g-C_3_N_4_, which was attributed to synergic effects at the ZnFe_2_O_4_/S-g-C_3_N_4_ interconnection. Antimicrobial activity of ZnFe_2_O_4_/S-g-C_3_N_4_ against Gram-positive and Gram-negative bacteria under visible light was performed. In addition, these ZnFe_2_O_4_/S-g-C_3_N_4_ NCs show a lot of promise as an antibacterial agent.

## Graphical Abstract

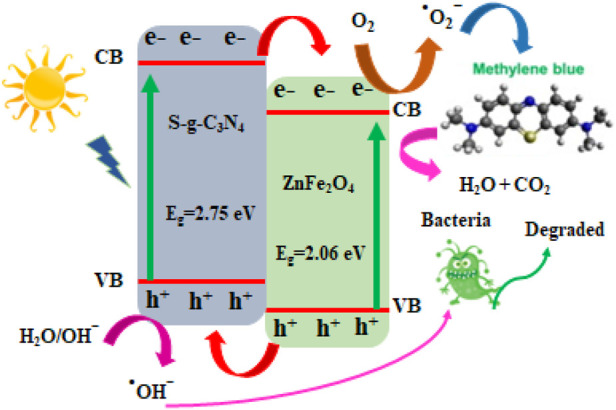

## Highlights

1) A binary ZnFe_2_O_4_/S-g-C_3_N_4_ heterojunction photocatalyst system is created *via* a Sol-gel technique.
2) An interfacial mediator between ZnFe_2_O_4_ and S-g-C_3_N_4_ works as an e^−^ and h^+^ separation mediator.
3) The ZnFe_2_O_4_/S-g-C_3_N_4_ heterojunctions’ photocatalytic and antibacterial properties were enhanced.

## 1 Introduction

Water is essential for living organisms, and owing to uncontrolled population growth and water pollution, there has been a subsequent rise in the scarcity of potable water. It entails that water-related problems must be addressed for the prosperous inhabitation of the living organism. In addition to population growth, industries have also contributed to the pollution of natural water bodies by introducing various pollutants (Liu et al., 2017; Qin et al., 2017; Sethi et al., 2019). Among these pollutants, Methylene blue (MB) is a representative pollutant affecting aquatic and terrestrial bodies (Habibi-Yangjeh and Shekofteh-Gohari, 2017; Shi et al., 2017; Qamar et al., 2020a). The development of technologies for removing domestic and industrial pollutants has become the centre of attention. In this regard, conventional water treatment methods such as filtration, adsorption and chlorination etc., are not recommended due to time-consumption, health hazards, cost, and may also lead to secondary pollution (Aoudj et al., 2015; Awual et al., 2015; Ali et al., 2018). Thus, the spotlight has been spotted on developing new resources to decontaminate water.

Due to the efficiency and efficacy of the advanced oxidation process (AOPs), which was first developed to clean drinking water, it is now used to treat wastewater (O’Shea and Dionysiou, 2012). Among various types of AOPs, heterogeneous photocatalysis is getting more attention (Syafiuddin et al., 2017; Iqbal et al., 2020a; Iqbal et al., 2020b; Iqbal et al., 2020c; Iqbal et al., 2021a). Semiconductor-based photo-catalysts are considered greener, more sustainable sources of reducing water pollution and other problematic outcomes (Sher et al., 2021a; Javed et al., 2021). All credit goes to low cost, chemical and biological inertness, and superb ability to remove organic contaminants, mainly dyes and toxins contained by wastewater (Kuo and Liao, 2006; Jan Šíma, 2013; Akhundi and Habibi-Yangjeh, 2016; Qamar et al., 2021a). Catalysis reaction is driven by light irradiation (UV light, solar light, visible light sources, etc.) on semiconductor catalysts. In heterogeneous photocatalysis, charge carriers are generated in sunlight, which in turn is followed by free radicals’ production. On further reactions, it generated free radicals eventually produced and mineralized pollutants into CO_2_ and H_2_O (Sahoo et al., 2018; Mishra et al., 2019). Among all known agents, spinal structured ferrites are accessible for brilliant photocatalytic degradation of organic compounds (Patna ik et al., 2018; Behera et al., 2019). Ferric oxide (Fe_2_O_3_) materials are regarded as ferrites (Biglari et al., 2016). Such ferrites have enclosed some advantages such as spinel like visible light responsiveness, internal magnetic properties and the availability of numerous stable photo-active sites (Atif et al., 2006; Xue et al., 2007). Among various known ferrites, zinc ferrite (ZnFe_2_O_4_) nanoparticles (NPs) having a narrow bandgap of 1.9 eV have been found as a potential candidate to exhibit remarkable photocatalytic activity (Iqbal et al., 2022).

Due to the fast recombination of a photo-generated exciton, ZnFe_2_O_4_ cannot be used as a photo-catalyst singly until a modification is made. These modifications include the formation of nanocomposites with other metal oxides, noble metals, doping with different metals and nonmetals, or the construction of hetero-junction photo-catalysts, etc., and contribute to resolving problems to some extent (Song et al., 2015). The construction of hetero-junction photo-catalysts has been valued and improved photocatalytic activity by raising electric current at the interface and diminishing the recombination phenomena. This function is carried out by graphitic carbon nitride (g-C_3_N_4_), a metal-free photo-catalyst, that has gained interest in the fabrication of hetero-junctions owing to unique properties including excellent stability, faster charge transport and low fabrication cost (Dong et al., 2014; Chen et al., 2015; Kuriki et al., 2015; Liu et al., 2015; Wang et al., 2018; Liras et al., 2019; Qamar et al., 2021b; Sher et al., 2021b; Qamar et al., 2022).

Despite advantages, properties such as weak *π*-*π* conjugated structures, low conductivity, less visible light utilization and small surface area have limited practical applications of g-C_3_N_4_ (Zhang et al., 2016; Iqbal et al., 2020d). And require modifications to improve light absorption properties. These modifications include doping with metals such as sulphur/metal oxides, non-metals and so on (Ali and Gupta, 2006; Liu et al., 2010; Dong et al., 2012; Hong et al., 2012; Feng et al., 2014; Wang et al., 2015; Jiang et al., 2017; Sher et al., 2021c). K.K.Das et al. have synthesized poly-pyrrole sensitized ZnFe_2_O_4_/sulphur doped g-C_3_N_4_ n-n hetero-junction and studied degradation of Ciprofloxacin (Das et al., 2020). N. Ali et al. constructed alkaline and transition metal ferrite photo-catalysts (MgFe_2_O_4_, BaFe_12_O_19_, ZnFe_2_O_4_, CaFe_2_O_4_ and CuFe_2_O_4_), and among them, zinc ferrite (ZnFe_2_O_4_) exhibited the best degradation of MB under sunlight (Ali et al., 2019). To the best of our knowledge, nanocomposites (NCs) composed of ZnFe_2_O_4_/sulphur doped g-C_3_N_4_ (S-g-C_3_N_4_) have not been studied yet, and such a new catalyst has not been reported for photocatalytic applications.

In this current study, ZnFe_2_O_4_ NPs and ZnFe_2_O_4_ NPs doped with S-g-C_3_N_4_ NCs have been prepared using sol-gel and simple chemical mixing methodologies. Both constructed NPs ZnFe_2_O_4_ NPs and ZnFe_2_O_4_/S-g-C_3_N_4_ NCs have also been characterized using XRD, XPS, EDX, UV-vis, EIS, photocurrent response and FTIR characterization techniques. ZnFe_2_O_4_/S-g-C_3_N_4_ NCs were examined for photo-catalytic activity against a model pollutant dye, MB degradation, and investigated spectrophotometrically as a viable candidate for photo-catalytic use. Irradiation has been done using solar light as a light source.

## 2 Materials and methods

### 2.1 Chemicals used

Zinc nitrate hexahydrate (Zn(NO_3_)_2_.6 H_2_O, 99.1%), ferrous nitrate (Fe(NO_3_)_2_), Citric acid, ammonium hydroxide (NH_4_OH), sulfuric acid (H_2_SO_4_), Thiourea ((NH_2_)_2_CS, ≥ 99.1%), Methylene blue (C_16_H_18_N_3_SCl, ≥99.01%). Sigma Aldrich provided all compounds, which were utilized without additional purification.

### 2.2 Photo-catalyst synthesis

#### 2.2.1 Synthesis of pure ZnFe_2_O_4_ NPs

The sol-gel method was employed to prepare pure ZnFe_2_O_4_ NPs. For this purpose, 2.97 g of Zn(NO_3_)_2_ and 4.05 g of Fe(NO_3_)_2_ were mixed and dissolved using de-ionized water. Citric acid (6.31 g) was added to the mixture after it had been dissolved. With the addition of NH_4_OH, the pH of the resultant combination was kept constant at 5.4. After adjusting pH, the obtained mixture was continuously stirred at 60°C for about 2 h before being heated to 90°C. After heating at 90°C, a gel was formed because of water evaporation and gel was then subjected to calcination in furnace for about 2 h at 450°C in order to decompose citric acid. The resulting product was cooled to room temperature after heating. After cooling, the product was washed several times using 5% of 0.1 M H_2_SO_4_ and de-ionized water till pH of filtrate was neutralized followed by drying in an oven at temperature of 80°C.

#### 2.2.2 Synthesis of S-doped g-C_3_N_4_

Recipe to synthesize S-doped g-C_3_N_4_ was followed by taking 30 g thiourea separately in each of three ceramic crucibles covered with lids and introduced in a muffle furnace at 520°C temperature for about 2 h, being kept at 5 °C/min. After 2 hours, the yellowish-colored product was allowed to cool naturally at room temperature before being ground and collected in dried sample vials. After grinding, yellowish product was used to synthesize NCs and tested for photo-catalytic activity.

#### 2.2.3 Synthesis of series of ZnFe_2_O_4_ doped with S-doped g-C_3_N NCs

A simple chemical mixing methodology was used to create a series of NCs composed of ZnFe_2_O_4_ coupled with S-doped g-C_3_N_4_ having variable ratios of S-g-C_3_N_4_. In order to develop a sequence, variable ratios i.e., 10%, 30%, 50%, 70% and 90% S-g-C_3_N_4_ were dissolved individually in 100 ml de-ionized water and continuously stirred for the period of 2 h. After complete dispersion, 2.97 g of ZnFe_2_O_4_ and 4.04 g of Fe(NO_3_)_2_ were mixed and dissolved using de-ionized water which was then followed by adding 6.3 g citric acid by weight. Afterwards, pH of resulting mixture was maintained at 5.4 using NH_4_OH. Once pH was adjusted, obtained mixture was continuously stirred for 2 h at 60°C before being heated to 90°C. After heating at 90°C, a gel was obtained as a result of water evaporation and was then calcined in furnace at 450°C for 2 h in order to decompose citric acid. After calcination, the product was collected from furnace and cooled at room temperature and washed using 5% of 0.1 M H_2_SO_4_ and de-ionized water several times until filtrate was obtained with neutral pH. Once neutral pH was stabilized, filtrate was dried in an oven at 80°C and collected in clean sample vials before being tested for photo-catalytic performance.

### 2.3 Characterization techniques

As shown in the supporting information, different ways of characterizing have been used for different purposes.

### 2.4 Photocatalytic performance

ZnFe_2_O_4_ doped with S-doped g-C_3_N_4_ NCs were tested for photo-catalytic activity against Methylene blue degradation (MB). For this purpose, sunlight as a light source was used to study photo-degradation. To begin, nano-samples in an aqueous MB solution were positioned in dark for duration of 30 min in order to establish adsorption-desorption equilibrium before being spectrophotometrically studied. The dye solution containing nano-samples in a Petri dish was then exposed to open atmosphere in the presence of sunlight. The pH of the following photo-catalytic reaction process is kept at its optimal value of 8. In the experiment, 0.01 g of ZnFe_2_O_4_/S-g-C_3_N_4_ NC was dissolved in 100 ml of MB aqueous solution. The resulting solution was then placed in a Petri dish in the dark mode for 30 min before being exposed to sunlight without a lid for dye photo degradation. After 30 min, 5 ml of MB solution was withdrawn, centrifuged and monitored through UV-vis spectrophotometer.

## 3 Results and discussion

### 3.1 Physicochemical properties of S-g-C_3_N_4_, ZnFe_2_O_4_ NPs, and ZnFe_2_O_4_/S-g-C_3_N_4_ NCs

#### 3.1.1 XRD analysis

**FIGURE 1:**
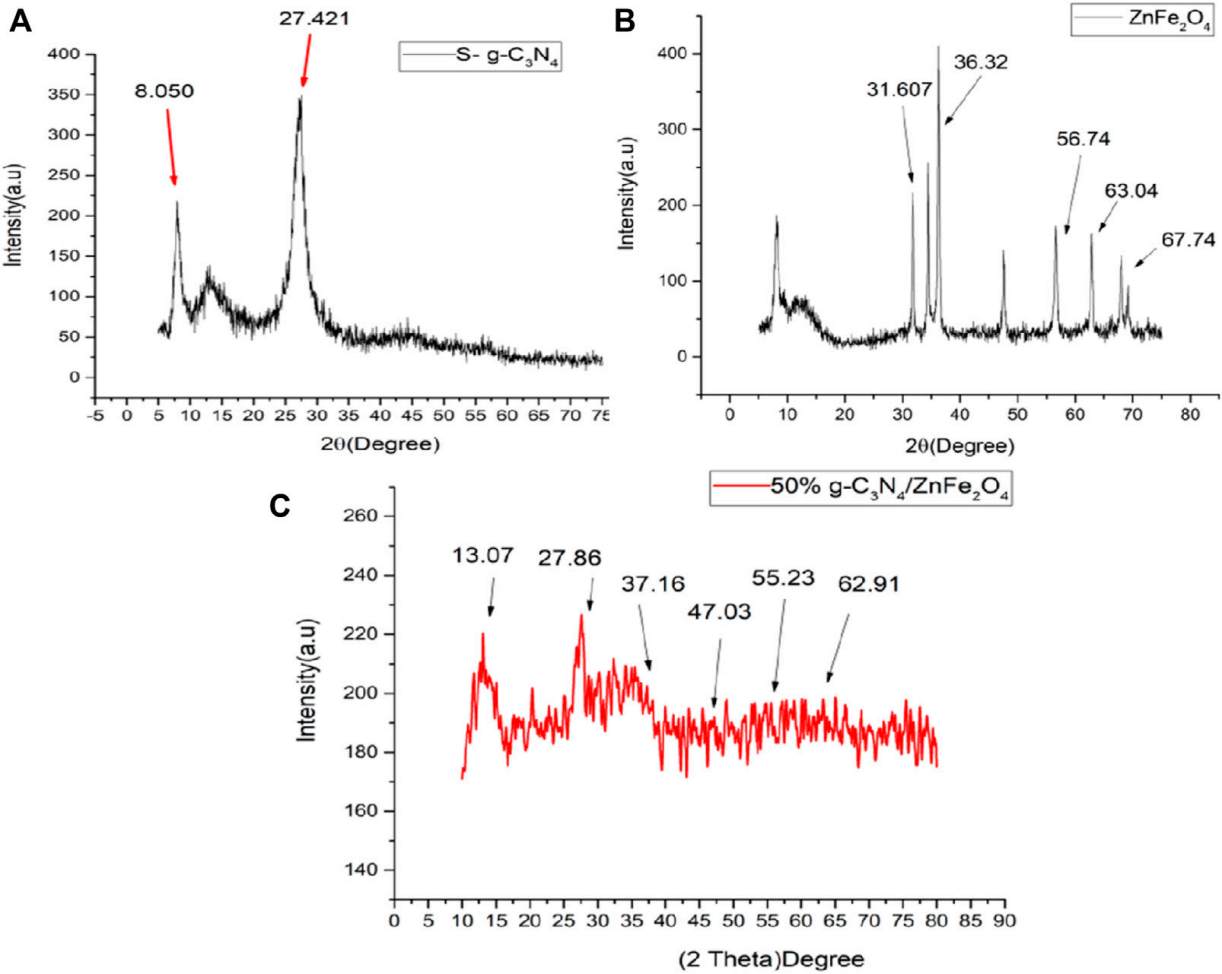
XRD pattern of **(A)** S-doped g-C_3_N_4_, **(B)** ZnFe_2_O_4_ NPs **(C)** 50% ZnFe_2_O_4_/S-g-C_3_N_4_ NCs.

XRD characterization technique was exercised to characterize phase composition of S-g-C_3_N_4_ at temperatures ranging from 0 to 80^o^ for prepared samples. Figure 1A depicts XRD pattern of S-g-C_3_N_4_. The sample has a single major peak (002) at 2 = 27.421^o^, indicating interlayer stacking of the matching aromatic conjugated system and confirming the synthesis of S-g-C_3_N_4_. Following the discovery of S doping in the z-orientation, the peak at 27.421^o^ indicates assembly of corresponding nano-sheets in the XY-plane. The peak at 8.050^o^ indicates a stronger H-bonding network, resulting in more in-plane order, due to the tri-s-triazine ring atoms (Jourshabani et al., 2017). Figure 1B shows the XRD pattern of ZnFe_2_O_4_ powder synthesized by sol-gel technique. The observed diffraction peaks are found to be in accordance with typical spinel structure of ferrite and are well defined by sharp increasing peaks, confirming the sample’s crystallinity. Diffraction peaks for a pure sample were obtained at 31.609°, 36.34°, 56.73°, 63.03° and 67.75°, which are linked to plane scattering, namely (220) (311) (511) (440) and (531). These scattering confirms the ZnFe_2_O_4_ powder’s face-centered cubic spinel structure (Pradeep et al., 2011; Rachna et al., 2018). XRD pattern of 50% ZnFe_2_O_4_/g-C_3_N_4_ NCs is shown in Figure 1C. Intensities of diffraction peaks are observed to be reduced and shifted which correspond to distinctive peaks at 13.08°, 27.87°, 37.17°, 47.04°, 55.25° and 62.92° and confirms successful formation of ZnFe_2_O_4_/S-g-C_3_N_4_ NC.

#### 3.1.2 TEM analysis

**FIGURE 2:**
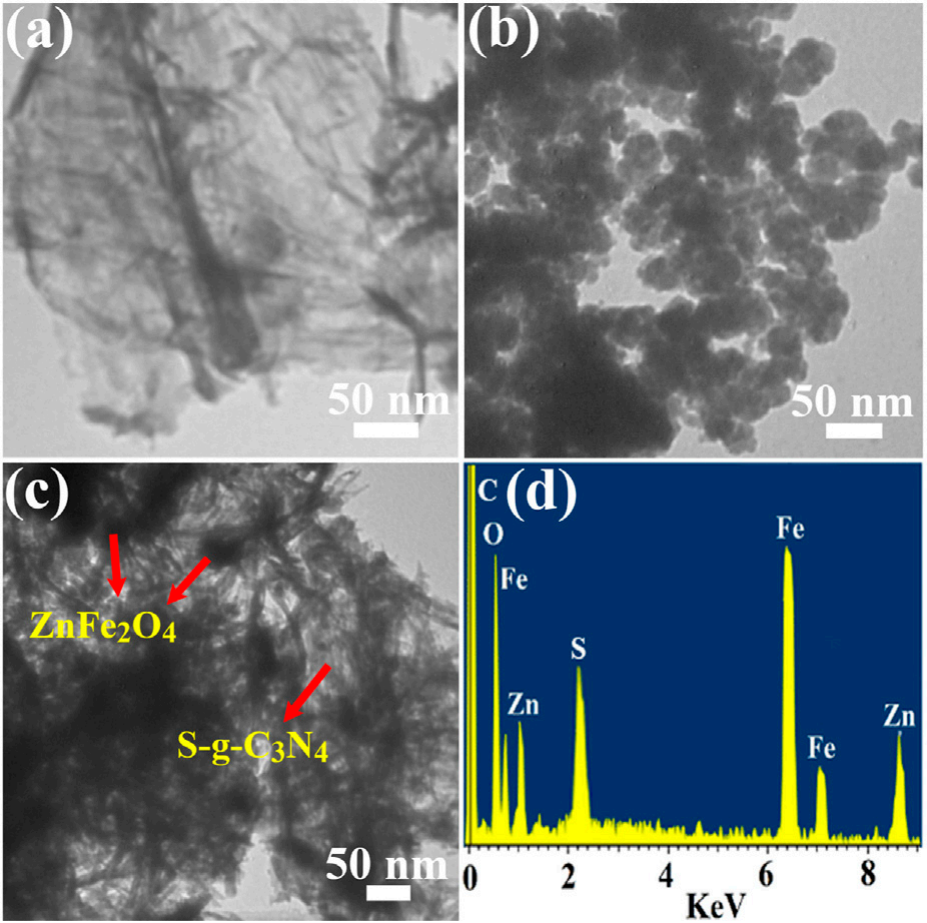
TEM images of **(A)** S-doped g-C_3_N_4_, **(B)** ZnFe_2_O_4_ NPs **(C)** 50% ZnFe_2_O_4_/S-g-C_3_N_4_ NCs and **(D)** shows the EDX of 50% ZnFe_2_O_4_/S-g-C_3_N_4_ NCs.

S-g-C_3_N_4_, ZnFe_2_O_4_ NPs, and ZnFe_2_O_4_/S-g-C_3_N_4_ NCs were investigated using TEM and EDX, respectively, to determine their crystalline structure, surface morphology, and elemental composition. As shown in Figure 2A, pure S-g-C_3_N_4_ possesses a two-dimensional (2D) nanosheets-like form with pronounced flexibility and aggregation. Figure 2B shows a scanning electron microscope picture of virgin ZnFe_2_O_4_ nanoparticles (NPs). With particle diameters ranging from 30–45 nm, ZnFe_2_O_4_ NPs are spherical, monodispersed, and have irregular shapes. Furthermore, the TEM picture (Figure 2C) indicates an equal distribution of ZnFe_2_O_4_ NPs across the S-g-C_3_N_4_ NSs in the case of 50 percent ZnFe_2_O_4_/S-g-C_3_N_4_ NCs. The S-g-C_3_N_4_ segment is a layered 2D structure with ZnFe_2_O_4_ scattered as 0D NPs.

When 50 percent S-g-C_3_N_4_ NSs were employed for production on ZnFe_2_O_4_ NPs, a well-defined heterojunction between ZnFe_2_O_4_ and S-g-C_3_N_4_ was found. The ZnFe_2_O_4_ and S-g-C_3_N_4_ heterointerface connection was shown to have an excellent interface contact in the TEM image (Figure 2C). The hetero contacts of ZnFe_2_O_4_ and g-C_3_N_4_ (Figure 2C) were found to be substantially integrated, which explains the composite systems’ considerable enhancement in photocatalytic ability. EDX elemental mapping of 50 percent ZnFe_2_O_4_/S-g-C_3_N_4_ NCs was also performed, as shown in Figure 2D, to analyze its surface component metal element. Carbon, iron, sulphur, oxygen, nitrogen, and zinc all had sharp peaks, suggesting that they were evenly distributed over the ZnFe_2_O_4_/S-g-C_3_N_4_ NCs.

#### 3.1.3 FT-IR spectrum analysis of ZnFe_2_O_4_ NPs and ZnFe_2_O_4_/S-g-C_3_N_4_ NCs (10%, 30%, 50%)

**FIGURE 3:**
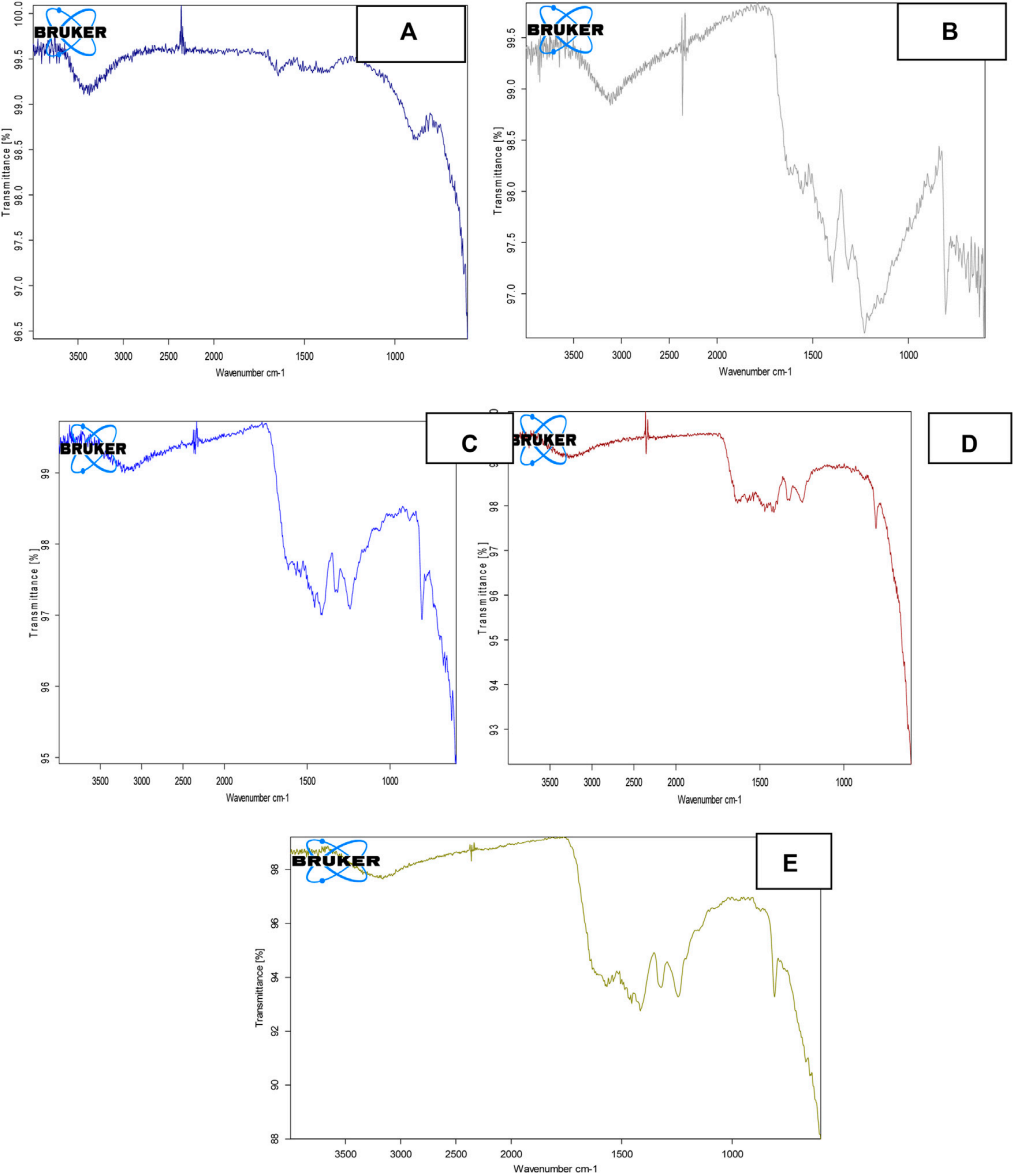
FT-IR spectra of **(A)** ZnFe_2_O_4_ NPs **(B)** S-g-C_3_N_4_ **(C)** 10% ZnFe2O4/S-g-C3N4 NC, **(D)** 30% ZnFe2O4/S-g-C3N4 NC, **(E)** 50% ZnFe2O4/S-g-C3N4 NCs.

The FT-IR spectrum of pure ZnFe_2_O_4_ NPs is displayed in Figure 3A. The obtained spectrum revealed two distinct absorption peaks being observed at 540-545 and 390-395 cm^−1^. These peaks were discovered to be associated with vibrational modes corresponding to oxygen-metal cation complexes at tetrahedral and octahedral sites, respectively. Because cations at both tetrahedral and octahedral sites are in different ionic states, the tetrahedral site has a wide shoulder and secondary bands. The above-mentioned band observation has also been aided by the intrusion of Zn^2+^ ions to the B-site, followed by Fe^3+^ ions to the A-site (Das et al., 2020). Figure 3B depicts the FT-IR spectrum for S-doped g-C_3_N_4_ ranging from 500 to 4,000 cm^−1^. In the measured spectra, a peak at 800–802 cm^−1^ highlights the vibrational frequency of triazine in condensed CN heterocycles, which is typical of triazine. Peaks in the 1,250–1,600 cm^−1^ range emphasize C-N aromatic ring stretching vibrations, whereas a large peak at 3,095–3,098 cm^−1^ is attributed to OH vibrations caused by the water molecule. This peak is also connected with the amino group’s N-H bond stretching vibrations. Peaks in the 1,200–1,050 cm^−1^ range were discovered, indicating S-doping. S-g-C_3_N_4_ synthesis is indicated by the measured spectrum and supporting evidence (Shcherban et al., 2016).

Figures 3C–E depicts the FT-IR spectra of 10%, 30 % and 50% ZnFe_2_O_4_/S-g-C_3_N_4_ NCs, respectively. Broad-spectrum in the range 3,100–3,500 cm^−1^ is observed for 10%, 30% and 50% ZnFe_2_O_4_/g-C_3_N_4_, confirming the presence of both amines (primary and secondary) and OH groups being adsorbed at surface. A number of peaks in the 1,250–1,600 cm^−1^ range have also been observed which indicate stretching vibrations due to C-N aromatic rings. The peak at 800 cm^−1^, which is typical of triazine and heptazine rings, shifts to a higher wave number, 805 cm^−1^, showing that ZnFe_2_O_4_ and S-g-C_3_N_4_ have a strong chemical connection. Water molecule vibrations are responsible for the large peak at 3,095–3,098 cm^−1^. A new peak in the region of 1,200–1,050 cm^−1^ is seen, confirming the existence of adequate sulphur and considerable ZnFe_2_O_4_/S-g-C_3_N_4_ NCs interaction, notably for 50 percent ZnFe_2_O_4_/S-g-C_3_N_4_ NCs. The acquired spectra validate the generation of pure ZnFe_2_O_4_ NPs, 10%, 30%, and 50% ZnFe_2_O_4_/S-g-C_3_N_4_ NCs, as well as the previously reported supporting results.

#### 3.1.4 XPS analysis

The elemental structure and valence state of 50 percent ZnFe_2_O_4_/S-g-C_3_N_4_ NCs were also determined using XPS. The peaks in the Zn 2p spectra of 50 percent ZnFe_2_O_4_/S-g-C_3_N_4_ NCs (Supplementary Figure S1A) attributable to the Zn 2p_3/2_ and Zn 2p_1/2_, correspondingly, may be assigned to the Zn 2p_3/2_ and Zn 2p_1/2_ (Khosravi-Gandomani et al., 2014; Zhu et al., 2020). In the Fe 2p XPS measurements (Supplementary Figure S1B), the oxidation state of Fe^3+^ in the constructed photocatalyst was assigned to two primary peaks of the Fe 2p_3/2_ (708.39) and Fe 2p_1/2_ (722.09) eV (Abdel Messih et al., 2019). The deconvoluted O 1s observations of 50 percent ZnFe_2_O_4_/S-g-C_3_N_4_ NCs (Supplementary Figure S1C) confirm the presence of two definite peaks at binding energies (BEs) of 531.3 and 529.8 eV, which may be linked with Zn-O and Fe-O, correspondingly (Zhao et al., 2020). Supplementary Figure S2 shows the C 1s spectrum. In the N 1s high-resolution spectra, the N C-N, N-H, and C(C)_3_ functions are attributed three characteristics peaks at 397.86, 400.64, and 399.76 eV, respectively (Supplementary Figure S1D) These XPS data showed that 50 percent ZnFe2O4/S-g-C3N4 NCs were successfully formed. The XPS findings showed that the contact between ZnFe2O4 and S-g-C3N4 is close, resulting in a nanocomposite containing 50% ZnFe2O4 and 50% S−g-C3N4.

#### 3.1.5 UV-vis analysis

The UV-vis spectra were used to assess the light absorption of the produced photocatalysts S-g-C_3_N_4_, ZnFe_2_O_4_, and 50 percent ZnFe_2_O_4_/S-g-C_3_N_4_ NCs. Supplementary Figure S3A shows a collection of UV-vis absorption spectra in the wavelength range of 285–752 nm. Light harvesting improves from 285 nm to 752 nm when 50 percent ZnFe_2_O_4_/S-g-C_3_N_4_ NCs are compared to ZnFe_2_O_4_ and S-g-C_3_N_4_. The integration of S-g-C_3_N_4_ NPs with S-g-C_3_N_4_ helps to increase the photocatalytic efficiencies of the 50 percent ZnFe_2_O_4_/S-g-C_3_N_4_ NCs material, which in turn helps to improve the photocatalytic efficiencies. Furthermore, the light-harvesting performance in the 450–752 nm region has been significantly increased, which is important for photocatalytic efficiency.

The energy bandgap values of these produced photocatalysts were determined by plotting UV-vis light-harvesting spectra using the Tauc’s plot (Supplementary Figure S3B). S-g-C_3_N_4_, ZnFe_2_O_4_, and 50 percent ZnFe_2_O_4_/S-g-C_3_N_4_ NCs were estimated to have bandgap values of 2.06 eV, 2.75 eV, and 2.27 eV, correspondingly, as shown in Supplementary Figure S3B. When the energy bandgap of S-g-C_3_N_4_ compared to ZnFe_2_O_4_ and 50 percent ZnFe_2_O_4_/S-g-C_3_N_4_ NCs declined from 2.75 eV for S-g-C_3_N_4_ to 2.27 eV for 50 percent ZnFe_2_O_4_/S-g-C_3_N_4_ NCs. The calculated energy bandgap of 50 percent ZnFe_2_O_4_/S-g-C_3_N_4_ NCs was 2.27 eV. The reduction in bandgap values might be attributable to the effective surface combination of both components, which significantly increases the binary photocatalytic capabilities. The photocatalytic capacities of ZnFe_2_O_4_ and S-g-C_3_N_4_ may be linked by the reduced optical bandgap edge of 50 percent ZnFe_2_O_4_/S-g-C_3_N_4_ NCs under visible light radiance.

### 3.2 Photo-catalytic activity of ZnFe_2_O_4_/S-g-C_3_N_4_ NCs

**FIGURE 4:**
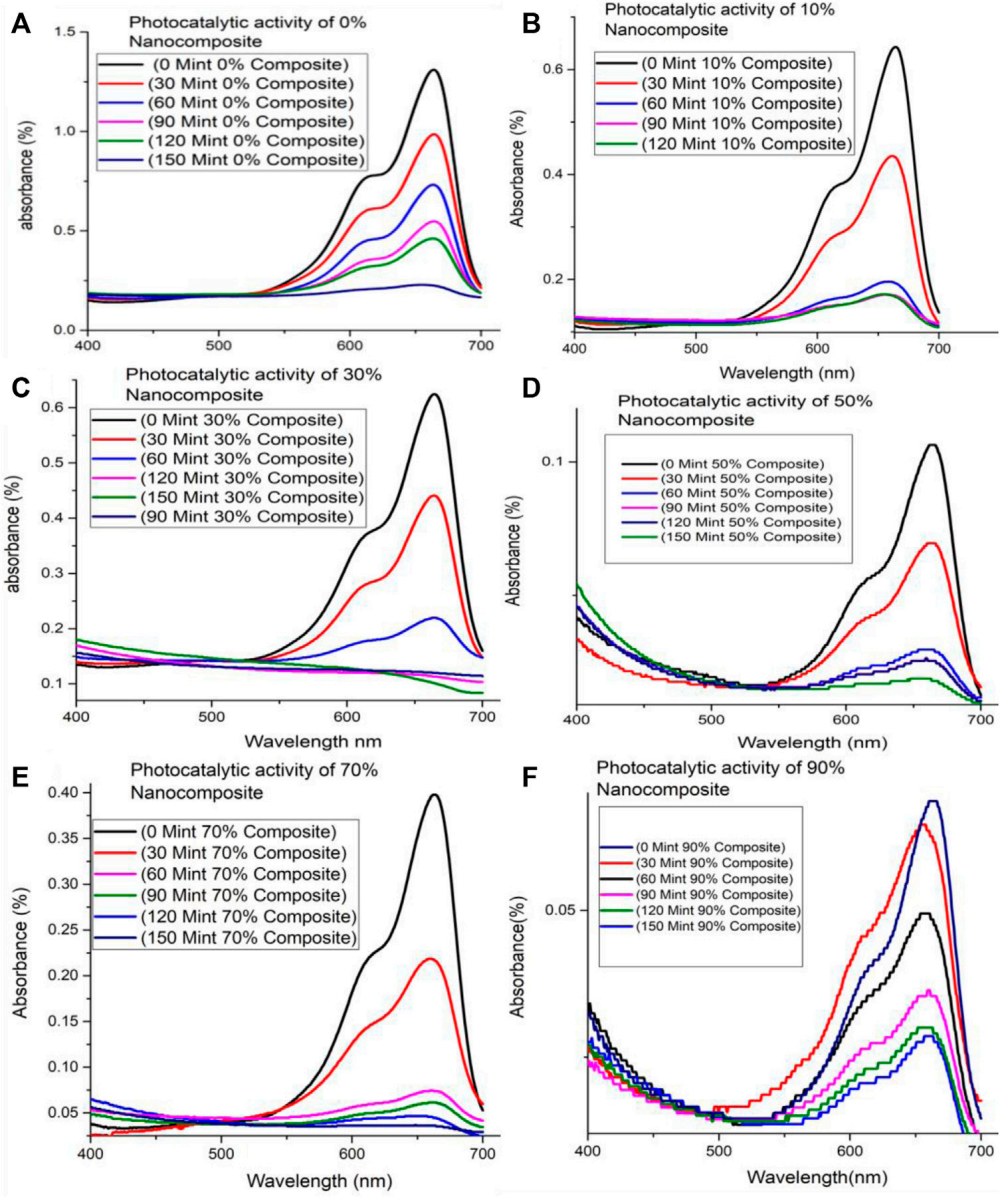
MB degradation under visible light illumination by **(A)** 0% ZnFe_2_O_4_/S-g-C_3_N_4_ NC **(B)** 10% ZnFe_2_O_4_/S-g-C_3_N_4_ NC **(C)** 30% ZnFe_2_O_4_/S-g-C_3_N_4_ NC **(D)** 50% ZnFe_2_O_4_/S-g-C_3_N_4_ NC **(E)** 70% ZnFe_2_O_4_/S-g-C_3_N_4_ NC **(F)** 90% ZnFe_2_O_4_/s-g-C_3_N_4_ NC.

Photo-activity of ZnFe_2_O_4_/S-g-C_3_N_4_ (10%, 30%, 50%, 70%, 90%) NCs has been tested for degrading dye i.e., methylene blue (MB). Herein, the potential application of ZnFe_2_O_4_/s-g-C_3_N_4_ NCs has been examined by nominating model reaction, photo-degradation of dye (MB) under visible light (Figure 4). It has been observed that MB spectra present maximum absorption at the wavelength of 664 nm, which reveals no degradation in the absence of either photo-catalyst, i.e., ZnFe_2_O_4_/s-g-C_3_N_4_ or visible light and verifies the stability of MB. On the other hand, photodegradation of MB is observed to start when MB solutions consisting of photo-catalysts ZnFe_2_O_4_/s-g-C_3_N_4_ being dispersed in solution are irradiated to sunlight. In the beginning, the photodegradation rate is speedy while starting to decay with time. Figures 4A–F displays photo-degradation in 150min associated with ZnFe_2_O_4_/s-g-C_3_N_4_ NCs with varying s-g-C_3_N_4_ amounts (10%, 30%, 50%, 70% and 90%).

**FIGURE 5:**
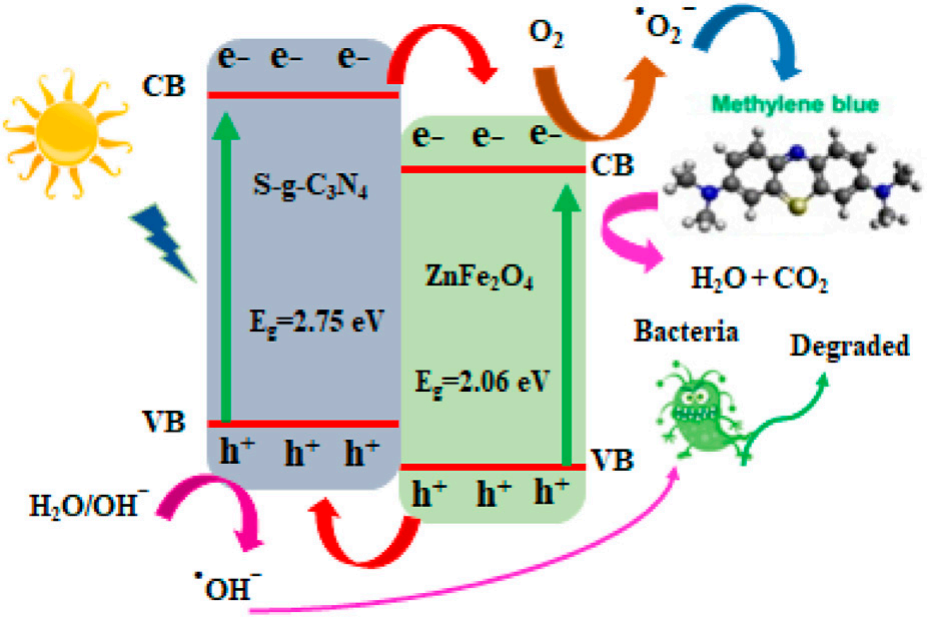
A hypothetical schematic diagram depicting a viable approach for photocatalytic dye decolorization using 50% ZnFe_2_O_4_/S-g-C_3_N_4_ NCs.

Comparative study of MB photo-degradation of ZnFe_2_O_4_/s-g-C_3_N_4_ NCs with varying S-g-C_3_N_4_ (0%, 10%, 30%, 50%, 70% and 90%) amounts has also been executed and presented in Supplementary Figures S4A–F. It has been observed from the results of MB photo-degradation by ZnFe_2_O_4_/s-g-C_3_N_4_ NCs that increased amounts of S-g-C_3_N_4_ resulted in enhanced MB’s degradation rate. The observed maximum photo-degradation rate has been achieved with 50 wt% S-g-C_3_N_4_ loading under sunlight. In addition to this observation, it has also been found that further increasing S-g-C_3_N_4_ amounts resulted into decreased photo-degradation activity (Qamar et al., 2020b; Iqbal et al., 2021b). Therefore, the 50 wt% s-g-C_3_N_4_ is called to exhibit efficient MB degradation performance and is applicable for delivering photo-induced electron-hole pairs separation across the 50% ZnFe_2_O_4_/s-g-C_3_N_4_ NCs interface. Increased amounts of S-g-C_3_N_4_ resulted in increased photo-degradation to some extent after that has been found to decline the dye photo-degradation performance. The creation of charge recombination centers or the light-blocking effect caused by large levels of S-g-C_3_N_4_ are credited. When 50% ZnFe_2_O_4_/S-g-C_3_N_4_ NCs are lighted by visible light, Figure 5 depicts the photocatalytic reaction pathways.

**FIGURE 6:**
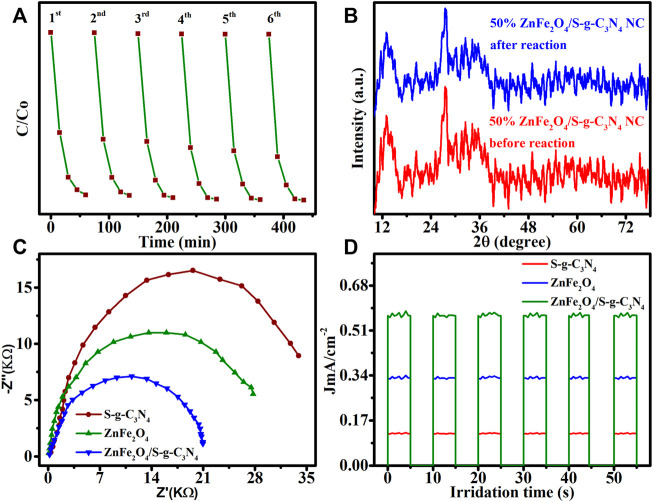
**(A)** Cyclic stability of 50% ZnFe2O4/S−g-C3N4 NCs photocatalyst for 6 sequential MB photodegradation tests. **(B)** Structural stability of 50% ZnFe2O4/S-g-C3N4 NCs identified by XRD patterns recorded before the first cycle and after the six-recycling test. **(C)** EIS Nyquist plots of S-g-C_3_N_4_, ZnFe2O4 and 50% ZnFe2O4/S-g-C3N4 NCs. **(D)** Transient photocurrent responses of undoped S-g-C_3_N_4_, ZnFe2O4 and 50% ZnFe2O4/S-g-C3N4 NCs in visible-light irradiation (*λ* > 420 nm).

The photo-decolorization of MB reusing samples with 50 percent ZnFe_2_O_4_/S-g-C_3_N_4_ NCs was tested six times under visible-light irradiation, as it is widely known that chemical stability of a catalyst is significant when deciding on its capacity for general application. The dye decolorization ability reduces somewhat after six runs (Figure 6A), suggesting that the NCs photocatalyst is chemically durable across a variety of experimental methodologies. The first and sixth dye photo-decolorization cycles yielded XRD patterns of 50 percent ZnFe_2_O_4_/S-g-C_3_N_4_ NCs, as illustrated in Figure 6B. Before and after MB recycling testing, 50 percent ZnFe_2_O_4_/S-g-C_3_N_4_ NCs showed no visible crystal phase structure changes, demonstrating chemical structural resilience. We think that when exposed to visible light, 50% ZnFe_2_O_4_/S-g-C_3_N_4_ NCs are extremely stable and dynamic catalysts, based on the findings of the research.

EIS in the dark was utilized to determine the heterointerface charge transfer rate at the electrode-electrolyte junction. A narrow arc radius is often related with lower electron transport resistance and faster interfacial photoinduced charge transfer and departure efficiency. The charge-transmission resistance of the 50 percent ZnFe_2_O_4_/S-g-C_3_N_4_ NCs was the lowest of all the synthesized samples (Figure 6C), indicating that the heterointerface contact of the 50 percent ZnFe_2_O_4_/S-g-C_3_N_4_ NCs can significantly support electron transmission, improving photocatalytic efficiency by increasing electron consumption. The results of the EIS are supported by transient photocurrent measurements. According to the aforementioned experimental results, a 50 percent ZnFe_2_O_4_/S-g-C_3_N_4_ NCs heterojunction may significantly improve heterointerface electron transmission, efficient separation of photogenerated e^−^ and h^+^ couples, and light harvesting capability.

We investigated the basis for 50 percent ZnFe_2_O_4_/S-g-C_3_N_4_ NCs with remarkable photocatalytic efficacy for MB photo-decolorization when equated to ZnFe_2_O_4_ and S-g-C_3_N_4_, which may explore the capable passage of photoinduced e^−^ and h^+^ pairs (Figure 6D). In a 0.5 M Na_2_SO_4_ solution, photocurrent density reactions of ZnFe_2_O_4_, S-g-C_3_N_4_, and 50 percent ZnFe_2_O_4_/S-g-C_3_N_4_ NCs were carried out under chopping illumination at 0 V. Under identical reaction conditions, the photocurrent response of the 50 percent ZnFe_2_O_4_/S-g-C_3_N_4_ NCs is considerably boosted, indicating that charge transfer and consumption are effective in this system. Photocurrent measurements show that photocatalytic MB destruction is aided by excellent electron-hole pair separation and refined heterointerfaces in self-assembled produced ZnFe_2_O_4_/S-g-C_3_N_4_ NCs.

### 3.3 Antibacterial ability

S-g-C3N4, ZnFe2O4, and 50 percent ZnFe2O4/S-g-C3N4 NCs were all tested independently for their antibacterial properties. The antibacterial capacity was tested using *S. aureus* (Gram-positive bacteria), *E. coli* (Gram-negative bacteria), *B. subtilis,* and *S. salivarius* bacteria as substrates. The positive and negative controls in these studies were double DI water and ciprofloxacin (0.6 mg/ml). Supplementary Table S1 and Supplementary Figure S5 show the results. The photocatalyst made up of 50% ZnFe2O4/S-g-C3N4 NCs had the greatest antibacterial ability, as predicted. Its large surface area and decreased e-/h + recombination propensity may be attributed to this. The production of reactive oxygen species (ROS) and its interaction with microorganisms are linked to the photocatalyst’s antibacterial capabilities. The significant antibacterial activity was attributed to the high production of ROS by the reaction of e-/h + formation by photocatalysts through chemisorption of water and oxygen. Under visible light irradiation, the 50 percent ZnFe2O4/S-g-C3N4 NCs shown good antibacterial efficacy against Gram-negative bacteria (*E. coli*) and Gram-positive bacteria (*B. subtilis, S. aureus, S. salivarius*).

## 4 Conclusion

The sol-gel methodological technique was used to successfully synthesis ZnFe_2_O_4_ NPs and ^ZnFe2O4/S−g-C3N4 NCs^ with various concentrations of S-g-C_3_N_4_ (10%, 30%, 50%, 70%, and 90%). Characterization methods such as XRD, TEM, EDX, EIS, XPS, photocurrent response and FT-IR were employed to ensure that the synthesis of NPs and NCs was successful. The potential application of prepared NCs, i.e., ^ZnFe2O4/S−g-C3N4 NCs^ (10%, 30%, 50%, 70 % and 90%), has been tested through photocatalytic performance against model dye (MB). A comparison of MB photo degradation by ^ZnFe2O4/S−g-C3N4 NCs^ with varying S-g-C_3_N_4_ (0%, 10%, 30%, 50%, 70% and 90%) amounts was also carried out, and it was discovered that 50 percent ^ZnFe2O4/S−g-C3N4 NCs^ displayed excellent photo-catalytic performance among all prepared NCs. In addition, decreased activity was observed with increased amounts of S-g-C_3_N_4_ owing to the formation of light-blocking effects. Degradation of wastewater pollutants, especially dyes, by NCs, i.e., ZnFe_2_O_4_/S-g-C_3_N_4_ as photo-catalyst, highlights attractive candidates.

## Data Availability

The original contributions presented in the study are included in the article/Supplementary Material, further inquiries can be directed to the corresponding authors.
