# Supplementary material for: Boosted spatial charge carrier separation of binary ZnFe2O4/S-g-C3N4 heterojunction for visible-light-driven photocatalytic activity and antimicrobial performance
Source: Front Chem. 2022 Aug 5;10:975355. doi: 10.3389/fchem.2022.975355 (PMC9388728; doi:10.3389/fchem.2022.975355)
Supplement: Supplementary file 1 [file DataSheet1.PDF]

Boosted Spatial Charge Carrier Separation of Binary $ZnFe_2O_4$/S-g-C_3N_4 Heterojunction for Visible-light-driven Photocatalytic activity and Antimicrobial Performance

Shahid Iqbal[1]*, Adnan Amjad[2,3], Mohsin Javed[3], M Alfakeer[4], Muhammad Mushtaq[2]*, Sameh Rabea[5], Eslam B. Elkaeed[5], Rami Adel Pashameah[6], Eman Alzahrani[7], Abd-ElAziem Farouk[8]

[1]*Department of Chemistry, School of Natural Sciences (SNS), National University of Science and Technology (NUST), H-12, Islamabad, 46000, Pakistan.*

[2]Department of Chemistry, Government College University, Lahore.

[3]Department of Chemistry, School of Science, University of Management and Technology, Lahore.

[4]Department of Chemistry, College of Science, Princess Nourah bint Abdulrahman University, P.O. Box 84428, Riyadh 11671, Saudi Arabia.

[5]*Department of Pharmaceutical Sciences, College of Pharmacy, AlMaarefa University, Riyadh 13713, Saudi Arabia.*

[6]*Department of Chemistry, Faculty of Applied Science, Umm Al-Qura University, Makkah 24230, Saudi Arabia.*

[7]*Department of Chemistry, College of Science, Taif University, P.O. Box 11099, Taif 21944, Saudi Arabia.*

[8]*Department of Biotechnology College of Science, Taif University, P.O. Box 11099, Taif 21944, Saudi Arabia.*

***To whom corresponding should be addressed**

shahidgcs10@yahoo.com (Shahid Iqbal) and Muhammad.mushtaq@gcu.edu.pk (Muhammad Mushtaq)

2.3. Characterization techniques

The crystalline structure of samples was recorded on D8, Burkur powder X-ray diffractometer ranging from 20-80° with a scan rate of 0.2^o s^{-1} by using Cu Kα radiations (1.5406 A^o), applied current of 40 mA in steps of 0.040 and accelerating voltage of 40 KV. The surface morphologies of the photocatalysts were determined employing a transmission electron microscope (TEM, JEOL-JEM-1230). The functional group analysis of samples was carried out by Alpha Bruker-Rays Fourier transform infrared spectrophotometer in the range of 4000-400 cm^{-1}. The degradation efficiency of samples was analyzed by a UV-visible spectrophotometer (Shimadzu, UV-1700) with a wavelength of 200-800 nm. XPS assessments were conducted on a Kratos Axis-Ultra multifunctional X-ray spectrometer. All BEs were consistent relative to C1s peak (284.8 eV) of the surface adventitious C to precisely the shift formed by the charge effect. Transient photocurrent response tests were evaluated on a standard three-electrode system (CHI 602 Electrochemical Workstation) at 25 ^{o}C with the Pt wire as the counter electrode, photocatalyst-coated FTO as the working electrode and Ag/AgCl as a reference electrode. The

EIS was assessed at -0.6 V (vs. Ag/AgCl) from 10^5 to 0.1 Hz with a signal amplitude of 20 mV.

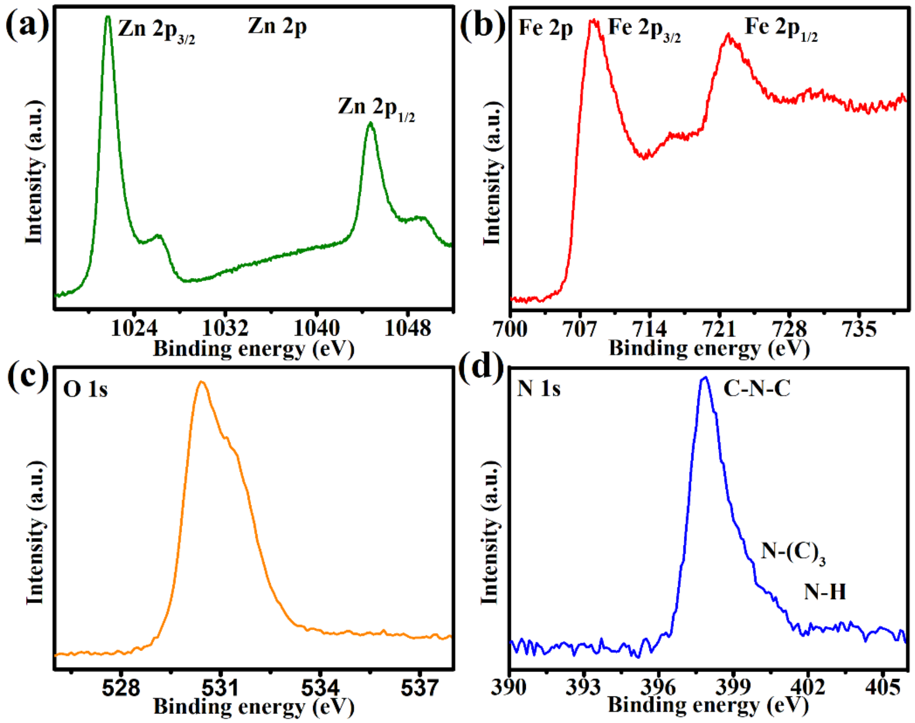

Figure S1. High-resolution XPS spectra of 50% $ZnFe_2O_4$/S-g-C_3N_4 NCs; (a) Zn 2p, (b) Fe 2p, (c) O 1s and (d) N 1s.

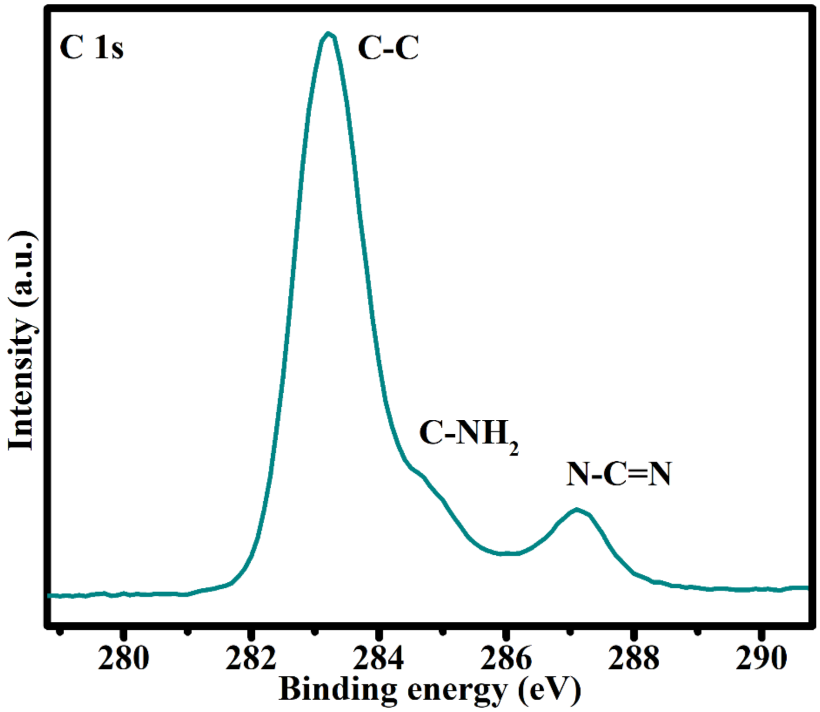

Fig. S2. High-resolution XPS C 1s spectra of 50% $ZnFe_2O_4$/S-g-C_3N_4 NCs.

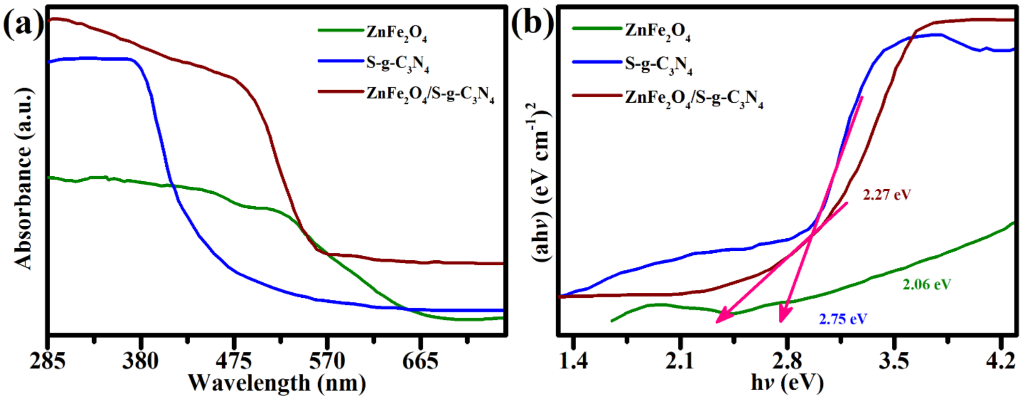

Fig. S3. (a) UV-vis absorption ranges and (b) Tauc's plots of S-g-C_3N_4, $ZnFe_2O_4$ and 50% $ZnFe_2O_4$/S-g-C_3N_4 NCs.

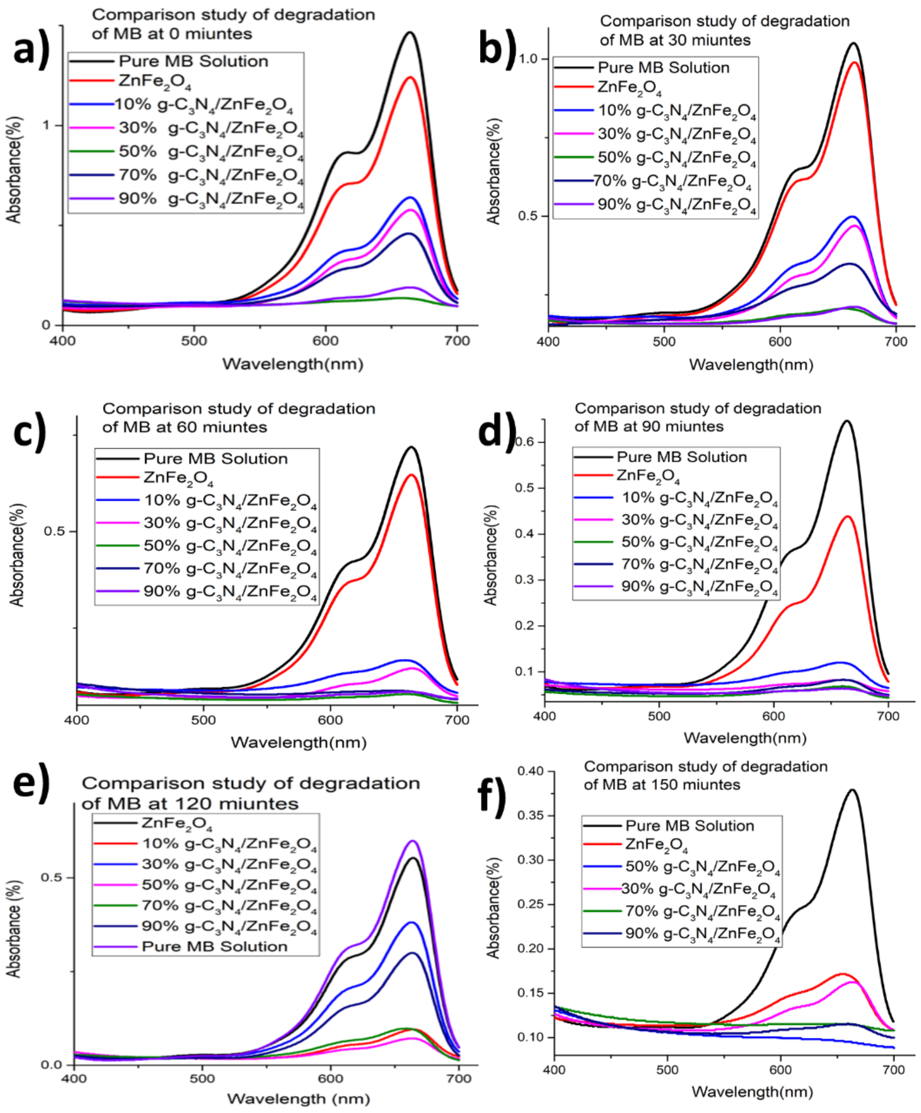

Fig. S4. Comparison of MB degradation under visible light illumination by 0%, 10%, 30%, 50%, 70% and 90% $ZnFe_2O_4$/s-g-C_3N_4 NC after (a) 0 min (b) 30 min (c) 60 min (d) 120 min (e) 150 min.

Table S1. Bactericidal Efficiency of S-g-C_3N_4, $ZnFe_2O_4$ and 50% $ZnFe_2O_4$/S-g-C_3N_4 NCs.

Antimicrobial agent	Escherichia Coli (mm)	Bacillus subtilis (mm)	Streptococcus salivarius (mm)	Staphylococcus aureus (mm)
Negative control	00	00	00	00
Positive control	16	17	20	15
S-g-C_3N_4	5.9	3.8	6.4	5.4
$ZnFe_2O_4$	9.2	8.3	11.5	10.4
$ZnFe_2O_4$/S-g-C_3N_4 NCs	18.3	15.8	19.8	18.4

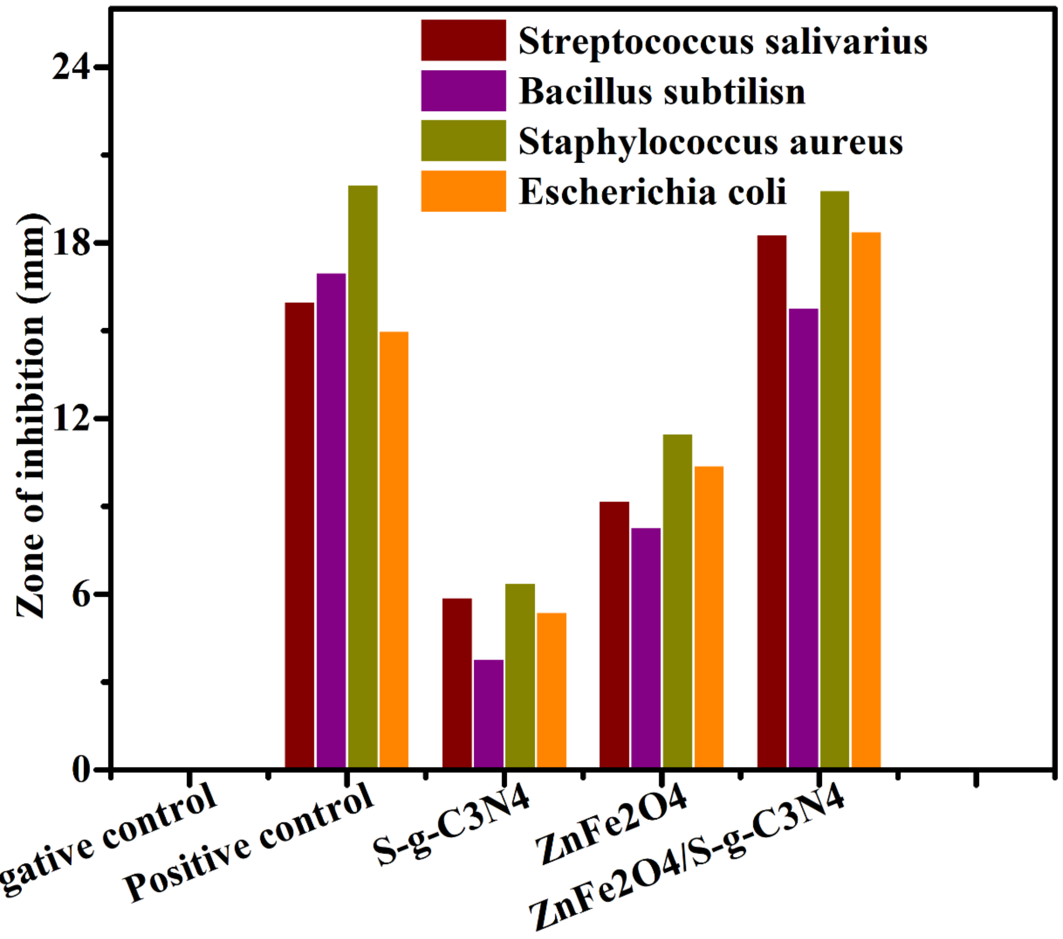

Fig. S5. Zone of inhibition (mm) of S-g-C_3N_4, $ZnFe_2O_4$ and 50% $ZnFe_2O_4$/S-g-C_3N_4 NCs against Staphylococcus aureus, Streptococcus salivarius, Bacillus subtilis and Escherichia Coli.
